# A comparative evaluation of dried activated sludge and mixed dried activated sludge with rice husk silica to remove hydrogen sulfide

**DOI:** 10.1186/1735-2746-10-22

**Published:** 2013-03-12

**Authors:** Seyed Mahmoud Mehdinia, Puziah Abdul Latif, Hassan Taghipour

**Affiliations:** 1 Department of Environmental Health, Damghan Faculty of Health, Semnan University of Medical Sciences, Semnan, Iran; 2 Department of Environmental Sciences, Environmental Studies Faculty, University Putra Malaysia, Serdang, Selangor Darul Ehsan, 43400, Malaysia; 3 Department of Environmental Health Engineering, Tabriz University of Medical Sciences, Tabriz, Iran

**Keywords:** Hydrogen sulphide, Rice husk silica, Dried activated sludge, Removal efficiency, Elimination capacity Pressure drop

## Abstract

The aim of this study was to investigate the effectiveness of dried activated sludge (DAS) and mixed dried activated sludge with rice husk silica (DAS & RHS) for removal of hydrogen sulfide (H_2_S). Two laboratory-scale filter columns (packed one litter) were operated. Both systems were operated under different conditions of two parameters, namely different inlet gas concentrations and different inlet flow rates. The DAS & RHS packed filter showed more than 99.96% removal efficiency (RE) with empty bed residence time (EBRT) of 45 to 90 s and 300 mg/L inlet concentration of H_2_S. However, the RE decreased to 96.87% with the EBRT of 30 s. In the same condition, the DAS packed filter showed 99.37% RE. Nonetheless, the RE was shown to have dropped to 82.09% with the EBRT of 30 s. The maximum elimination capacity (EC) was obtained in the DAS & RHS packed filter up to 52.32 g/m^3^h, with the RE of 96.87% and H_2_S mass loading rate of 54 g/m^3^h. The maximum EC in the DAS packed filter was obtained up to 44.33 g/m^3^h with the RE of 82.09% and the H_2_S mass loading rate of 54 g/m^3^h. After 53 days of operating time and 54 g/m^3^h of loading rates, the maximum pressure drop reached to 3.0 and 8.0 (mm H_2_O) for the DAS & RHS packed and DAS packed filters, respectively. Based on the findings of this study, the DAS & RHS could be considered as a more suitable packing material to remove H_2_S.

## Introduction

Waste gases containing reduced sulphur compounds, such as hydrogen sulfide (H_2_S), dimethyl sulphide (Me_2_S) and methyl mercaptan (MeSH), have an unpleasant odour even at extremely low concentrations [1]. H_2_S is irritating, smelly substance with very low odour threshold number up to 1.1 parts per billion [2]. This unwanted pollutant is emitted into the atmosphere from different industrial processes including leather manufacturing, food processing, livestock farming and wastewater treatment processes [3]. Commonly used processes of H_2_S treatment include chemical and physico-chemical methods. However, these methods have high operating costs and produce chemical waste by-products that must be disposed [4]. The application of biofiltration systems are more attractive because they are inexpensive and cause no environmental pollution [5-7]. Lately, the influence of even low concentrations of air pollutants on human health has re-emerged as an important scientific issue. Several studies have linked various acute and chronic health impacts to air pollution [8]. It is important to note that biological treatment system is one of the earliest biological processes which has been developed for the elimination of gaseous compounds and used for odor removal in the wastewater treatment industry. At present, the biological treatment system is becoming more popular because it is a green technology which does not use chemicals and also does not produce wastes which are potentially dangerous for the environment. This process is essentially based on the ability of micro-organisms to transform both organic and inorganic pollutants into less toxic and odorless compounds [9].

The main goal of this study was to investigate the removal efficiency of hydrogen sulphide using two packing materials, namely dried activated sludge and mixed dried activated sludge with rice husk silica. Moreover, elimination capacity and pressure drop versus operating time, different empty bed residence time (EBRT) and different inlet concentration of H_2_S was investigated.

## Materials and methods

### Characterization of the packing materials

Dried activated sludge and mixed rice husk silica with dried activated sludge were used as packing material for removal of H_2_S. Dried activated sludge with mixed microbial culture was collected from a sewage treatment plant (Putrajaya STP 2) in Malaysia. Rice husk silica was prepared according to Jamwal and Mantri’smethod. First of all, the rice husk was washed with tap water to clean the dirt. Then the washed rice husk was dried in an oven at 110°C for 24 h. The washed and dried rice husk was then subjected to acid leaching. This was done by reflux in 3% (v/v) hydrochloridric acid (HCL) and 10% (v/v) sulphuric acid (H_2_SO_4_) for two h, at a ratio of 50 g husk/L. Then, the rice husk was washed with distilled water and then dried in an air oven at 100°C for four h. After all, the cleaned husk was burned inside a muffle furnace at temperature of 800°C in a porcelain crucible for four h in static air [10]. Meanwhile, rice husk silica was mixed with dried activated sludge (50–50 volume) and used in the filter as packing material. Some of the important characteristics of the rice husk silica and the dried activated sludge are listed in the Table 1.

**Table 1:** Some of the important characteristics of dried activated sludge and rice husk silica used in this study

**Dried activated sludge**			**Rice husk silica**		
**Properties**	**Unit**	**Value**	**Properties**	**Unit**	**Value**
**Particles size:**	(Weight %)		**Physical properties:**		
< 2 mm		0	Density	(g/L)	52
2-4 mm		22	Surface area	(m^2^/g)	226.3
4-6 mm		24	Median pore radius	(nm)	2.3747
6-8 mm		21	Cumulative pore volume	(cm^3^/g)	0.3078
8-15 mm		33	**Chemical composition:**	(%)	
>15 mm		0	SiO_2_		97.35
**Density**		634	SO_3_		1.66
**Analysis of elements:**	(%)		K_2_O		0.43
C		3.56	**Analysis of elements:**	(%)	
H		6.98	C		0.05 ±0.01
N		0.45	H		0.27 ±0.01
S		0.043	N		0.36 ± 0.05

### Filters operation

**Figure 1:**
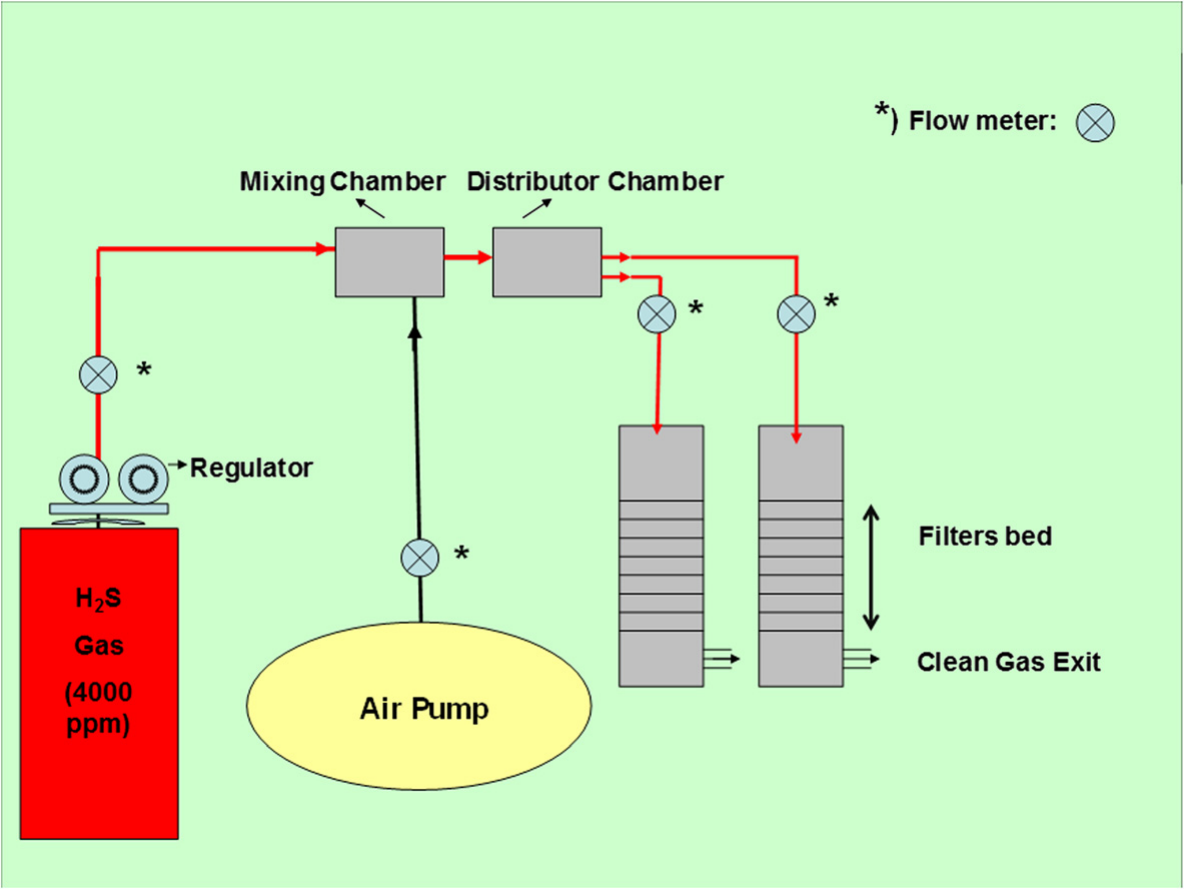
The schematic flow diagram of the pilot.

This research was a lab-scale study that was carried out in the laboratory at the Faculty of Environmental Studies at University Putra Malaysia. The flow diagram of this pilot is shown as a schematic diagram in Figure 1. Two filters were constructed using PVC cylinder with 50 cm in height and 7.5 cm in diameter (packed one liter). H_2_S gas cylinder with 4000 mg/L and 150 bars inside pressure was used. An air pump has been used to oxygen supply for aerobic bacteria in the filters and for dilution with gas to produce different concentration of H_2_S in mixing chamber. This study was carried out in two stages. In the first stage, the system was operated with varying inlet concentrations of H_2_S and a constant flow rate of 1.0 L/m. According to the study by Jeong et al. in order to adapt the micro-organisms in the filters, the tested H_2_S gas was introduced in the filters at low concentration of 10 mg/L for one week. After the adaptation period, H_2_S concentration at the inlet was increased in weekly increments [11], and this was from 10 mg/L to a final concentration of 300 mg/L (10, 50, 100 and 300 mg/L). In the second stage, the system was operated with varying flow rates (based on the different EBRT between 30 and 90 s (30, 45, 60, 75, 90 and 90 s) and a constant inlet concentration of 300 mg/L. Two filters were operated at the temperature of 25 ± 2°C.

### Data analysis and performance of the filters

In this study, inlet and outlet concentration of H_2_S of each filter were measured every day using hydrogen sulfide detector model ppb RAE 3000, U.S.A. removal efficiency (RE), elimination capacity (EC) of H_2_S and pressure drop (mm H_2_O) were used as indicators of the performance of filters as stated below:

(1) $$\mathrm{RE}\left[\%\right]=\left({\text{C}}_{\mathrm{Gi}}–{\text{C}}_{\mathrm{Go}}\right)/{\text{C}}_{\mathrm{Gi}}\times 100$$

(2) $$\mathrm{EC}\left[\text{g}/{\text{m}}^{3}\text{h}\right]=\left({\text{C}}_{\mathrm{Gi}}–{\text{C}}_{\mathrm{Go}}\right)\times \text{Q}/{\text{V}}_{\text{f}\;}$$

Where Q is the gas flow rate (m^3^/h), V_f_ is the volume of the filter bed (m^3^), C_Gi_ and C_Go_ are the inlet and outlet hydrogen sulfide concentration (mg/L) [12-15]. In the Faculty of Chemistry University Putra Malaysia the chemical analysis including analysis of elements (CHNS) and chemical composition was carried out. Moreover, Brunauer-Emmett-Teller (BET) specific surface area was performed by using a ThermoFinnigan Sorptomatic apparatus using nitrogen adsorption at −196°C for rice husk silica.

## Results

**Figure 2:**
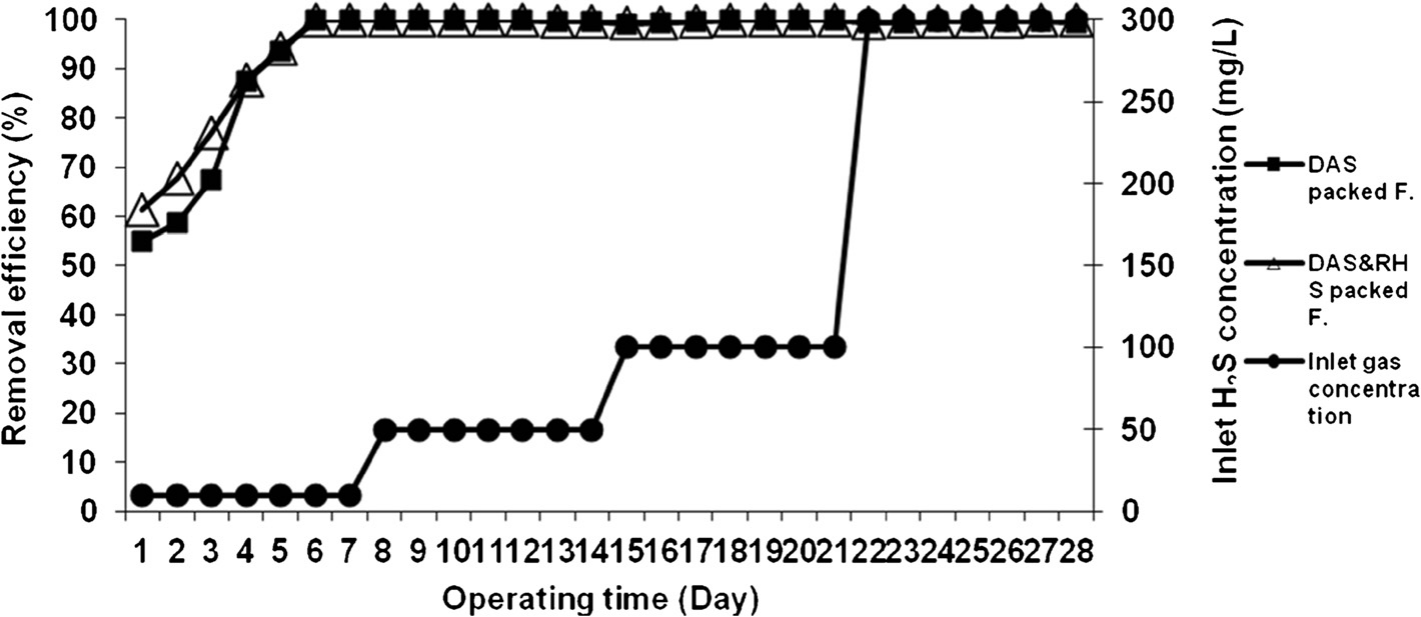
**Removal efficiency of H**_**2**_**S vs. operating time and different inlet concentrations of 10–300 mg/L and fixed EBRT of 60 s in the filters.**

In the first stage, the systems were operated with a low inlet concentration of H_2_S (10 mg/L). After five days of operating time, the removal efficiency of both packed filters reached the maximum amount of 100% (note that the adaptation period was five days). After adaptation period, removal efficiency with different inlet concentration of H_2_S from 10 to 300 mg/L in the dried activated sludge packed and the mixed rice husk silica and dried activated sludge packed filter was greater than 99.02% and 99.26%, respectively. Figure 2 shows the results of average removal efficiency (RE%) in the filters with a fixed flow rate (1.0 L/min) and different inlet concentration of H_2_S (10–300 mg/L) that increased weekly.

**Figure 3:**
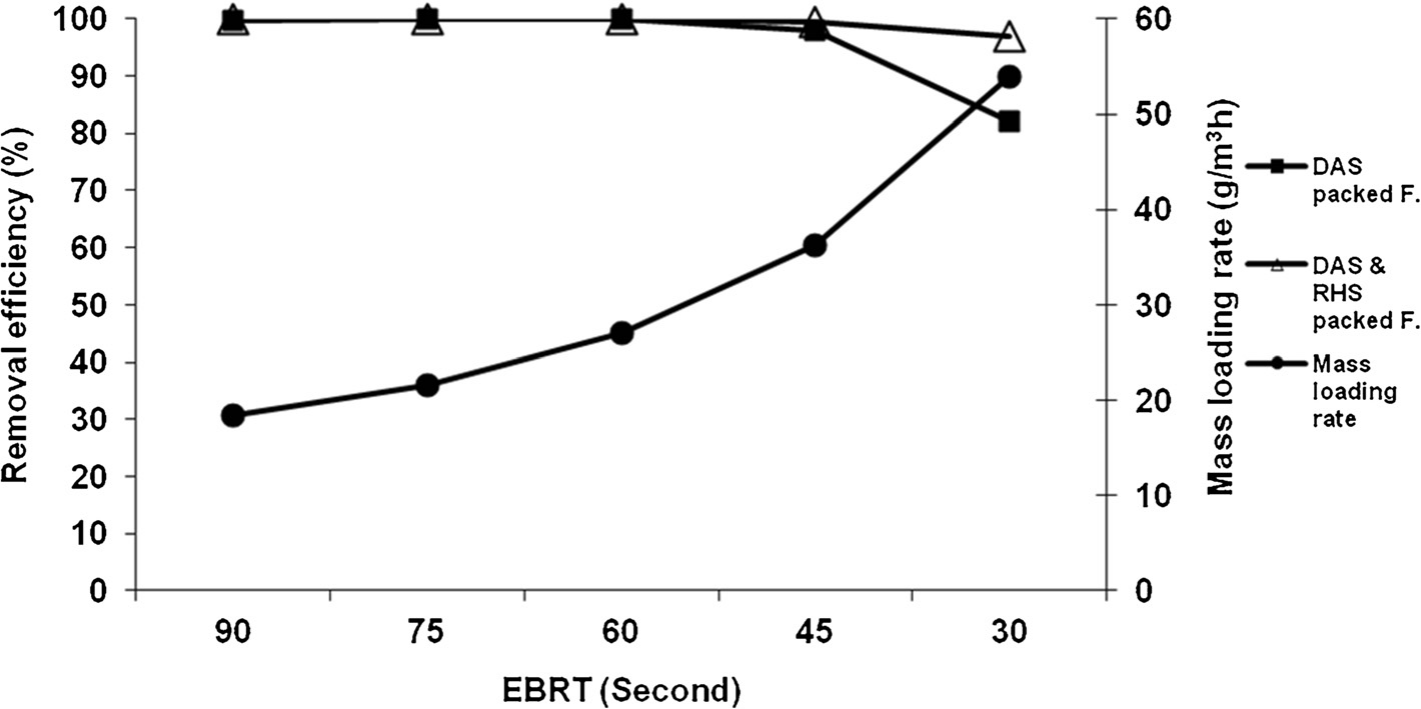
**Removal efficiency (%) vs. different EBRT (s) with different H**_**2**_**S mass loading rates (g/m**^**3**^**h) in the filters.**

The results for the removal efficiency (%) versus different empty bed residence times (EBRT) and different mass loading rates in the filters are shown in Figure 3.

**Figure 4:**
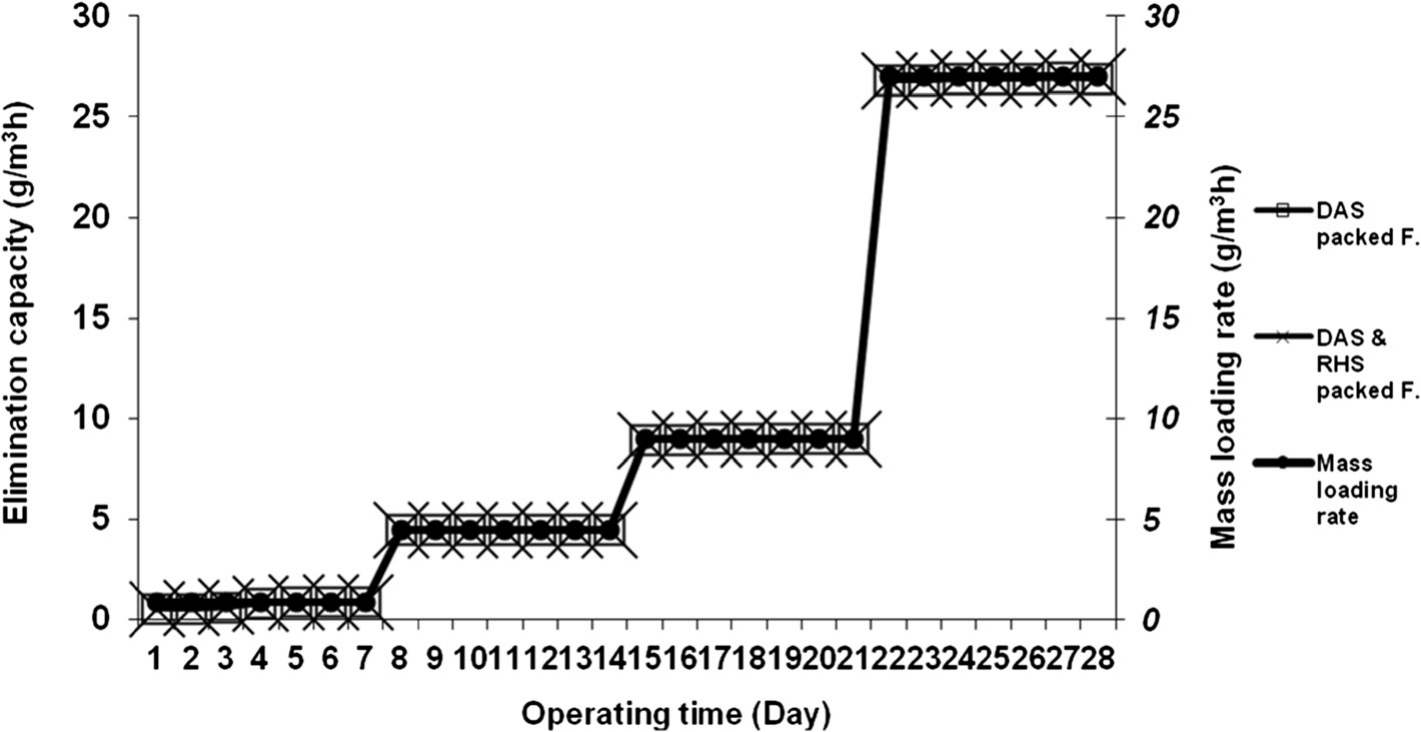
**Elimination capacity (g/m**^**3**^**h) vs. operating time (day) and different loading rates (g/m**^**3**^**h) at a constant EBRT of 60 s in the filters.**

The maximum elimination capacity (EC) was obtained in the filter packed with mixed rice husk silica with dried activated sludge up to 26.98 g/m^3^h, with the RE of 99.94% and H_2_S mass loading rate of 27 g/m^3^h. The dried activated sludge packed filter showed the maximum EC up to 26.84 g/m^3^h with the RE of 99.51% and H_2_S mass loading rate of 27 g/m^3^h. The results gathered for the EC versus operating time and the different inlet concentrations of H_2_S in the filters are shown in Figure 4.

**Figure 5:**
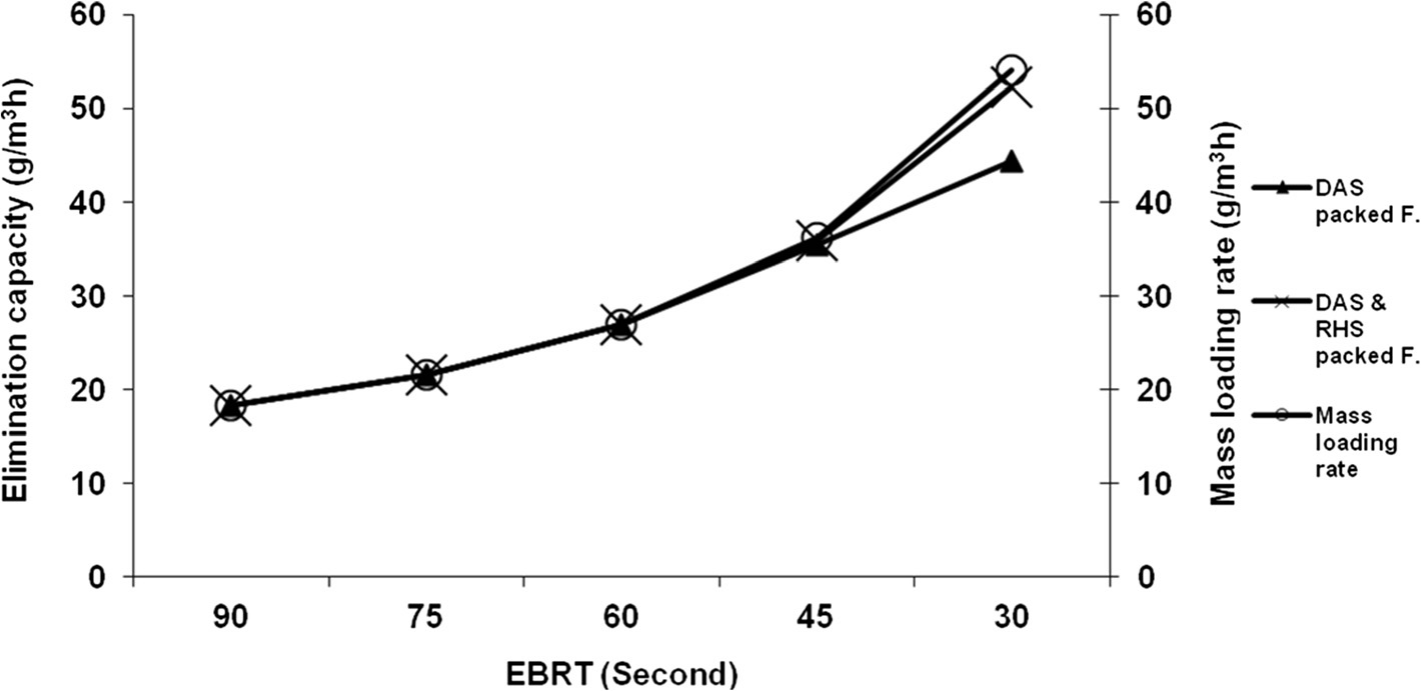
**Elimination capacity (g/m**^**3**^**h) vs. different EBRT (s) and different H**_**2**_**S mass loading rates (g/m**^**3**^**h) in the filters.**

The results for the elimination capacity (g/m^3^h) versus different empty bed residence times (EBRT) and different mass loading rates in the filters are shown in Figure 5.

**Figure 6:**
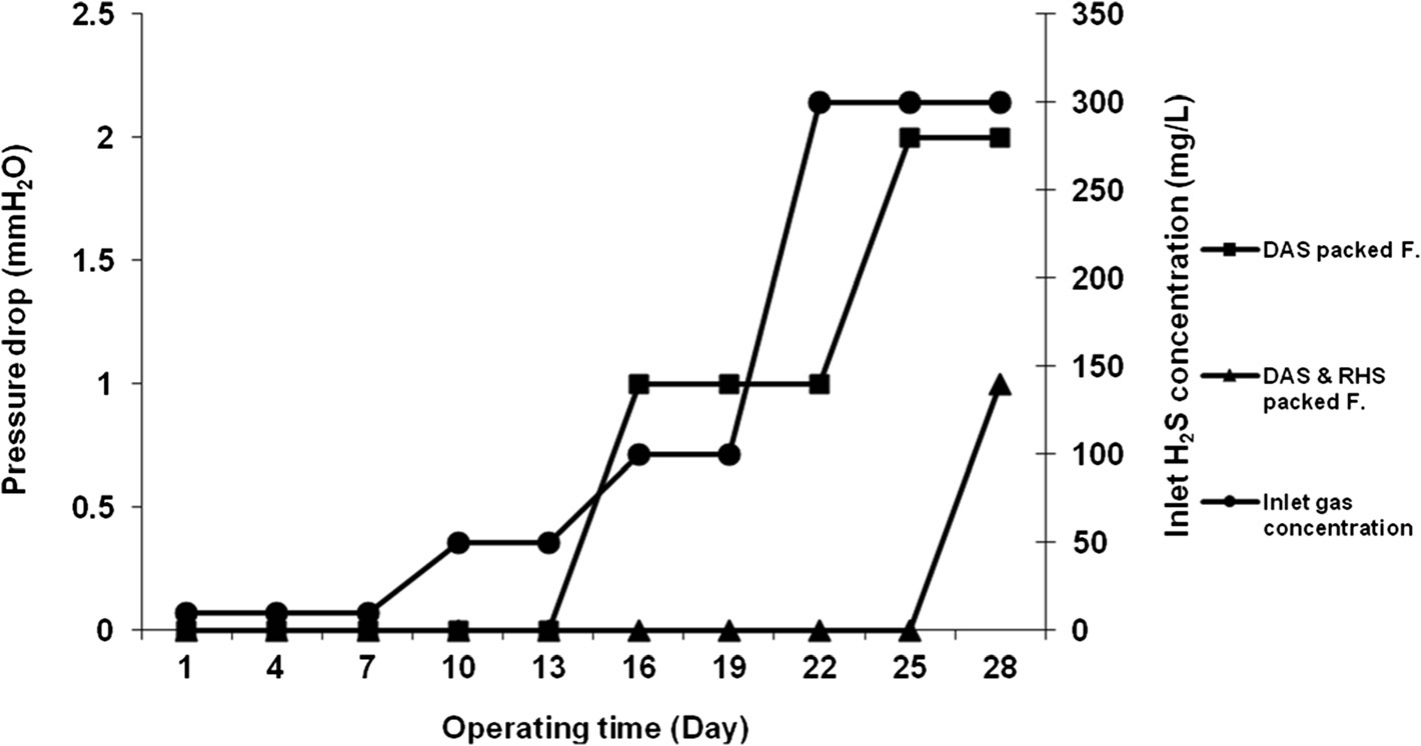
**The changes in pressure drop (mm H**_**2**_**O) vs. operating time (day) and different inlet concentration of H**_**2**_**S (ppm) in the filter.**

In the first stage of this study, the pressure drop in the filters was measured versus operating time and different inlet concentrations of H_2_S. Nevertheless, the pressure drop was in undetectable amounts in the two filters at the beginning of the operating time. After 28 days of operating time, as well as with the EBRT of 60 s and different inlet concentrations of H_2_S (from 10 to 300 mg/L), the pressure drop was found to have increased to 2.0 and 1.0 (mm H_2_O) in the dried activated sludge packed filter and the mixed rice husk silica with dried activated sludge packed filters, respectively. Figure 6 shows the amount of pressure drop changes versus operating time and different inlet concentrations of H_2_S in the filters.

**Figure 7:**
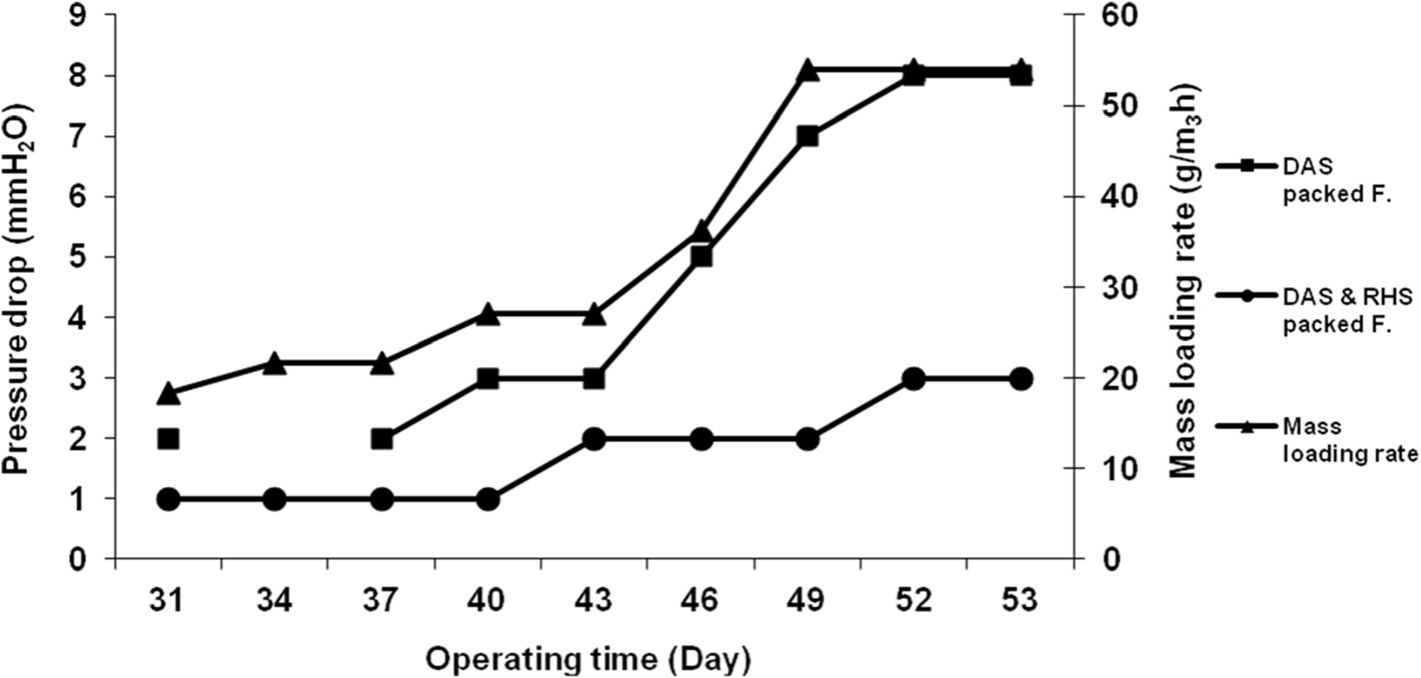
**The pressure drop changes (mm H**_**2**_**O) vs. operating time (day) and different loading rate (g/m**^**3**^**h) in the filters.**

In the second stage of this study, the amount of changes in the pressure drop (mm H_2_O) versus operating time (day) and different mass loading rates (g/m^3^h) were measured and the results are shown in Figure 7.

## Discussion

The mixed rice husk silica with dried activated sludge packed filter showed greater than 99.96% removal efficiency (RE) with the EBRT of 45 to 90 s and 300 mg/L inlet concentrations of H_2_S. However, its RE was found to have decreased to 96.87% with the EBRT of 30 s. The dried activated sludge packed filter showed 99.37% of RE in the same condition. The RE, nevertheless, dropped to 82.09% with the EBRT of 30 s. Masoudinejad et al. reported 90% of RE with an inlet H_2_S concentration up to 93.34 mg/L, after three weeks of operating system. They used Thiobacillus thioparus on the seashell bed biofilter [16]. Meanwhile, Lee et al. achieved stable RE of over 99%, with the EBRT ranging from 8.2 to 60 s at the 250 mg/L of H_2_S inlet concentration. They also found that the RE was reduced by about 10 percent when the retention time was reduced to 5.5 s. In their study, they used a biofilter packed with scoria, and inoculated with Bacillus sp. as H_2_S oxidizer [17].

The maximum EC was obtained in the mixed rice husk silica with dried activated sludge packed filter up to 52.32 (g/m^3^h) with the RE of 96.87% and the H_2_S mass loading rate of 54 (g/m^3^h). At the RE greater than 99.96%, however, the maximum EC was 26.99 (g/m^3^h) with H_2_S mass loading rate of 27 (g/m^3^h). The maximum EC in the dried activated sludge packed filter was obtained up to 44.33 (g/m^3^h) with the RE of 82.09% and H_2_S mass loading rate of 54 (g/m^3^h). Roshani et al. reported the maximum EC of about 22 g-S/m^3^ h for the biofilter during the operating time with the maximum inlet H_2_S concentration of 265 mg/L [18]. Meanwhile, Kim et al. recorded the maximum EC of 8 g H_2_S g/m^3^h at a loading rate of 13 g H_2_S g/m^3^h. In their study, they used a bio filter packed with biomedia, encapsulated by sodium alginate and polyvinyl alcohol (PVA) [19]. Ramirez et al. obtained a critical EC of 14.9 g/m^3^h with the RE of 99.8%. However, they found the maximum EC of 55.0 g/m^3^h with the RE of 79.8% and the EBRT of 150 s. The researchers also investigated the removal of H_2_S using immobilized Thiobacillus thioparus in a biotrickling filter packed with polyurethane foam [20].

In the mixed rice husk silica with dried activated sludge packed filter, the maximum pressure drop reached 3.0 mm H_2_O after 53 days of operating time and 54 g/m^3^h of mass loading rates. The maximum pressure drop in the dried activated sludge packed filter reached up to 8.0 mm H_2_O in the same condition. McNevin and Barford reported an increasing pressure drop from less than 500 to greater than 2500 pa after 3 months of continuous operating time [21]. Meanwhile, the maximum pressure drop of 18 mm H_2_O was reported in the study by Roshani et al. who evaluated the performance of biofiltration in the removal of H_2_S from gas stream [18].

## Conclusion

Higher performance (higher removal efficiency, higher elimination capacity, and lower pressure drop) was obtained in the filter packed with mixed rice husk silica and dried activated sludge. Therefore, based on the results of this study, mixed rice husk silica with dried activated sludge could be considered as more suitable packing material for removal of hydrogen sulfide.

## Competing interests

The authors declare that they have no competing interests.

## Authors’ contributions

The overall implementations of this study were the results of efforts by corresponding author. All authors have made contribution into the review and finalization of this manuscript. All authors read and approved the final manuscript.
